# Extended Adhesion-Sparing Liver Eversion during Kasai Portoenterostomy for Infants with Biliary Atresia

**DOI:** 10.3390/children8090820

**Published:** 2021-09-17

**Authors:** Filippo Parolini, Giovanni Boroni, Pietro Betalli, Maurizio Cheli, Domenico Pinelli, Michele Colledan, Daniele Alberti

**Affiliations:** 1 Department of Paediatric Surgery, “Spedali Civili” Children’s Hospital, 25123 Brescia, Italy; giovanni.boroni@unibs.it (G.B.); daniele.alberti@unibs.it (D.A.); 2 Department of Paediatric Surgery, ASST Papa Giovanni XXIII, 24127 Bergamo, Italy; pbetalli@asst-pg23.it (P.B.); mcheli@asst-pg23.it (M.C.); 3 Department of Surgery III, ASST Papa Giovanni XXIII, 24127 Bergamo, Italy; dpinelli@asst-pg23.it (D.P.); mcolledan@asst-pg23.it (M.C.); 4 Department of Clinical and Experimental Sciences, University of Brescia, 25123 Brescia, Italy

**Keywords:** biliary atresia, Kasai portoenterostomy, liver eversion, liver transplantation, bowel adhesions

## Abstract

Background: Despite the fact that Kasai portoenterostomy (KPE) is the primary treatment for biliary atresia (BA), liver transplantation (LT) remains the ultimate surgery for two-thirds of these patients. Their true survival rate with the native liver reflects the original KPE and the burden of post-operative complications. We report an original modification of the adhesion-sparing liver eversion (ASLE) technique during KPE that facilitates the total native hepatectomy at time of transplantation. Methods: All consecutive patients with BA who underwent KPE at our department and subsequent LT at Paediatric Liver Transplant Centre at Papa Giovanni XXIII Hospital between 2010–2018 were retrospectively enrolled. All patients underwent ASLE during KPE. Patients’ demographic data, type of KPE, total transplant time (TTT), hepatectomy time (HT), intra-operative packed red blood cells and plasma transfusions, intra- and post-operative complications were noted. Results: 44 patients were enrolled. Median TTT and HT were 337 and 57 min, respectively. The median volume of packed red blood cell transfusion was 95 mL. No patients presented bowel perforation during the procedure or in the short post-operative course. No mortality after LT was recorded. Conclusions: In addition to the well-known advantages of the standard liver eversion technique, ASLE reduces the formation of intra-abdominal adhesions, lowering significantly the risk of bowel perforation and bleeding when liver transplantation is performed for failure of KPE.

## 1. Introduction

Biliary atresia (BA) is an obliterative cholangiopathy of infancy, representing the leading indication for pediatric liver transplantation in the world and accounting for approximately 50% of transplants in children and 10% of transplants at any age [1,2]. In 1959, Kasai reported an operative procedure consisting of a hepatic portoenterostomy (KPE) in which the atretic extrahepatic tissue was removed, and a Roux-en-Y jejunal loop was anastomosed to the hepatic hilum in order to restore bile drainage [3]. Nowadays, this operation with current modifications is widely accepted and has become established as the primary treatment for BA [3,4], resulting in successful bile drainage in more than half of patients [1,4]. However, liver transplantation (LT) remains the ultimate surgery for BA and two-third of BA patients will require LT due to the progression of BA-related chronic liver disease [2,5,6,7]. The true survival rate of these patients reflects the success of the original KPE, the prevention of post-operative complications (such as bleeding, ascites, cholangitis and adhesions) and the quality of access and safety of the transplant procedure, with minimization of intra- and post-operative risks [4,5,6]. In this study we report our experience with an original extended adhesion-sparing liver eversion (ASLE) technique during KPE. Our modification of the standard technique reduces the formation of intra-abdominal adhesions and, at transplantation, eases the native liver total hepatectomy and decreases bleeding and the incidence of bowel perforations.

## 2. Materials and Methods

### 2.1. Population of the Study

All consecutive patients with histologically confirmed BA who underwent KPE at our department and subsequent LT at the Pediatric Liver Transplant Centre at Papa Giovanni XXIII Hospital between January 2010 and December 2018 were enrolled in this retrospective study. All KPEs were performed by one surgeon (DA) and all LTs were performed by two surgeons (MC, NP). A minimum of 1 year of follow-up after transplantation was available for each enrolled patient. The study was approved by our Institutional Review Board and Ethics Committee (IRB n° 3924/2020). A written consent was obtained from patients’ parents before all steps of the study. All data were retrospectively collected and recorded according to the Declaration of Helsinki.

### 2.2. Measures and Outcomes

For each enrolled patient, demographic, clinical and laboratory data and type of KPE were collected together with total transplant time (TTT), hepatectomy time (HT), packed red blood cells (PRBC) and plasma transfusion volumes, weight at transplantation, intra and post-operative complications occurring within the first month and overall survival at 1 year after LT. Actuarial survival rate was defined as the time from the date of surgery to the earlier date when the patient died (events) or withdrew or was lost at follow-up (censored). TTT was defined as the time between skin incision and abdominal wall closure. HT was defined as the period between skin incision and clamping for total liver exclusion. The requirement of blood transfusions has been calculated on the basis of plasma and PRBC volume administered during the transplant procedure.

### 2.3. Protocol

Standard KPE consists of resecting the atretic gallbladder and the extrahepatic biliary tree, leaving a denuded porta hepatis, which is then reconstructed into a 40-cm jejunal Roux loop (2–3). In the early 2000s, one of the authors (DA) developed this original technique for liver eversion, which was called the “extended” adhesion-sparing liver eversion (ASLE).

**Figure 1:**
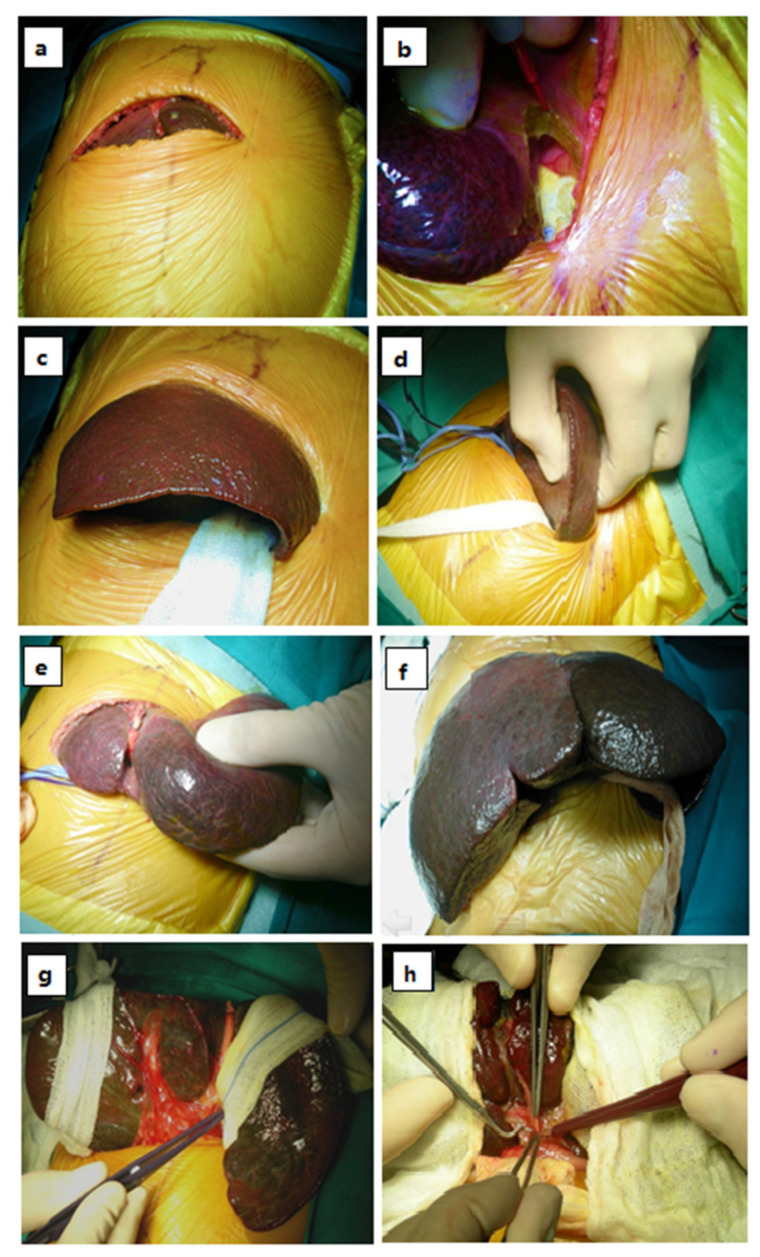
Laparotomy and ligation and division of the round ligament of the liver (**a**). Insertion of gauze between the left triangular and left coronary ligaments and the stomach and spleen. (**b**) The left lobe is gently grasped (**c**), is then gently rotated back to front (**d**) and is eventually pulled toward the left until achieving complete eversion (**e**). Complete eversion of the entire liver outside the laparotomy incision is accomplished by gently grasping the right lobe, which is pulled toward the left without section of the right ligaments (**f**). A swab is then placed in the hepatic loggia and two other gauzes, surrounding the left and right lobes, are cranially grasped to the drapes. At this point the liver is completely dislocated towards the outside, with the two gauzes keeping the liver well-everted, thus providing excellent exposure of the porta hepatis (**g**). The liver is then covered by two ribbon gauze soaked in saline solution to humidify the organ and to avoid air exposure in order to prevent the subsequent development of adhesions (**h**). At this time the bowel is completely kept inside the abdomen. Please note that even in an infant with severe hepatomegaly, the liver is safely exteriorized through a small laparotomy incision (compare Figure 1a,g)).

After a short skin incision and limited section of large abdominal muscles, the technique involves the section of the left triangular ligament and a limited division of the left coronary ligament only. A peculiar rotation of the liver enables the entire organ to be mobilized and totally exteriorized out of the abdomen. In addition to providing an excellent exposure of the portal structures, our ASLE prevents even the breakthrough of the small bowel from the abdominal cavity, reducing the formation of post-operative adhesions. After having confirmed the diagnosis of BA (minilaparotomy with or without intra-operative cholangiography), the steps of our original maneuver are the following (Figure 1).

First, we begin with the widening of the laparotomy (usually less than 8 cm length) from half of the left rectus muscle to the whole right rectus muscle (see Figure 1a). After tying and dividing the round ligament, we place a gauze under the left triangular and coronary ligaments, tacking care to protect the stomach and spleen, in order to avoid injuries to these during the division of the ligament with electrocautery (see Figure 1b). After resting a small gauze on the left liver lobe, segments II and III are gently grasped with hands (see Figure 1c); the left liver lobe is then gently rotated from back to front (see Figure 1d), and at the same time, it is pulled up, first out of the abdomen and then toward the left until achieving its eversion (see Figure 1e). Complete eversion of the entire liver outside the laparotomy incision is accomplished by gently grasping the right lobe (segments V and VIII) and, without dividing the right triangular and coronary ligaments, the right lobe is pulled out, again first toward the left side and then medially. To facilitate the right liver eversion, two retractors push down the right part of the surgical wound (see Figure 1f). A small gauze is then placed in the hepatic loggia and two other gauzes, surrounding the left and right lobes, are cranially grasped to the drapes. At this point the liver is completely dislocated toward the outside, with the two gauzes keeping the liver well-everted, thus providing excellent exposure of the porta hepatis without the use of any retractors that could injure the hepatic parenchyma (see Figure 1g). The liver is then covered by two ribbon gauzes soaked in saline solution to humidify the organ and to avoid air exposure in order to prevent the subsequent development of adhesions. In this way a complete, quick and safe eversion of the liver is accomplished. The following steps of the Kasai procedure, including the meticulous dissection of the hilum and preparation of the fibrous plate, are performed using the standardized technique [3]. During all these steps the bowel is completely kept inside the abdomen (see Figure 1h).

After dissecting the fibrous biliary remnants, the liver is then placed back into the abdominal cavity and a Roux-en-Y loop is created. A second liver eversion with the same previously described steps is required in order to transect the atretic bile ducts and perform the portoenteroanastomosis. A silicon abdominal drain is routinely placed in the supramesocolic space.

## 3. Results

In the time span of the study, 48 patients who had previously undergone standard KPE were transplanted for inadequate biliary drainage with biliary cirrhosis. Of them, 3 were excluded from the study because the transplant was managed in other institutions, and 1 was excluded because of incomplete follow-up. The remaining 44 patient were enrolled. The median age of patients at transplantation was 13.1 months (range 5–120) and median weight was 8.1 kg (range 5–23). Median TTT was 337 min (IQR = 272–371) and median HT was 57 min (IQR = 50–67). Median volume of intra-operative PRBC transfusion was 95 mL (IQR = 0–250) and median volume of plasma transfusion was 0 mL (IQR = 0–110). No patient presented with bowel perforation during the transplant procedure or in the early post-operative course. One year graft loss was 2.2% (1/44) and no mortality was recorded. These outcomes are summarized in Table 1.

## 4. Discussion

Although the KPE has dramatically improved the outcomes in children affected by BA, most of them eventually require LT, even after an initially successful KPE [7,8,9,10,11]. The original Kasai procedure presents an extremely crucial role, as many outcomes (including clearance of jaundice, survival with native liver and overall survival rate) of these patients are directly influenced by the experience of the surgeon and the nature and quality of the surgery performed. Previous series have outlined the improvement both in native liver survival and in outcomes of LT when the KPE has been performed in high-volume centres [12,13].

The original KPE consists of resecting the atretic gallbladder and the extrahepatic biliary tree, leaving a denuded porta hepatis, which is then reconstructed into a 40 cm jejunal Roux loop [2,4,6]. To facilitate the procedure, Valayer and colleagues proposed the exteriorization of the entire liver by dividing the falciform, coronary and triangular ligaments [14], and after their description, the technique was adopted by many other leading pediatric surgeons [4,6,15,16]. Chardot and colleagues in 2009 reported an additional improvement of this technique by dividing only the falciform and the left triangular ligaments [15]. Nevertheless, according to our experience, dividing the falciform ligament is not necessary. Furthermore, sectioning the right triangular and coronary ligaments requires a generous widening of the laparotomy, which facilitates the breakthrough of the small bowel from the abdominal cavity, increasing the occurrence of post-operative adhesions. We have found that the division of the left triangular ligament together with a limited section of the left coronary ligament only, followed by a peculiar maneuver, enables the entire liver to be mobilized and exteriorized up, providing excellent exposure of the portal structures and reducing the formation of diffuse adhesions encountered in case of subsequent liver transplantation. Just before the liver eversion, it is necessary to warn the anesthetist, as the maneuvers could impair venous return to the heart by kinking the inferior vena cava [3]. No ASLE-maneuver-related complications, such as bleeding and/or hepatic hematoma, were recorded. Even an enlarged liver, as usually occurs in BA infants, can be entirely everted through a small laparotomy. Our technique decreases the formation of inflammatory adhesions between the right liver and diaphragm, which is a common problem with the standard liver eversion. These adhesions, with the progression of cirrhosis, usually became the seat of hepatofugal collateral veins that, at transplantation, increase the risk of bleeding during the native liver total hepatectomy. Moreover, as the bowel is kept inside the abdomen for the near-total time of the KPE (Figure 1), this assures a minimal exposure of the intestinal loops, which significantly reduces the formation of bowel adhesions and decreases the occurrence of bowel perforations. Dense intestinal adhesions are frequently observed at the time of LT in recipients who have undergone previous KPE and intestinal perforations occur in up to 20% of cases [10,11,17,18,19]. Surgical bowel perforations, which can also occur in the first post-operative days, are associated with high rates of morbidity and mortality [10,19]. Therefore, the quality of the previous surgery is crucial, as intra-abdominal adhesions and prolonged operative LT time, which may be increased in case of diffuse adhesions, are well-known independent risk factors for bowel perforation [10].

Increased evidence supports the use of hyaluronate carboxymethylcellulose membrane (Seprafilm, Genzyme Corp., Cambridge, MA, USA) in pediatric patients undergone laparotomy [20,21]. Nevertheless, the advantages of this film during KPE have not been clearly established; in particular, it could prevent the formation of adhesions between the bowel and abdominal wall, but it scantly decreases the rate of intestinal adhesions. Moreover, the abdominal drain as a foreign body could possibly have been associated with intestinal adhesions in few reports in adult settings [22,23]. Nevertheless, in our experience we found that advantages of the use of a small, silicon drainage placed in the supramesocolic area clearly exceeded this potential risk; an abdominal drain is considered a standard practice in many European Centers after KPE [6].

Transfusions of blood products have been strongly associated with a worse outcome after LT in term of major infectious, cardiovascular, respiratory, and bleeding complications [24,25,26]. The amount of intra-operative blood product administered is crucial, as high-volume transfusion is associated with a significant decrease in both graft and patient survival [24,25]. Recently, Gordon and colleagues demonstrated in a large series of pediatric liver transplantation that high-volume transfusion (more than 27.5 mL/kg) increased the mortality risk by threefold compared to low-volume transfusion [24]. Although in our cohort it is clearly difficult to state with certainty whether the reduced need for intra-operative transfusions during LT was directly related to the liver-eversion maneuver at time of KPE, our findings strongly suggest this is somewhat related to the quality of the original surgery.

Recently, the laparoscopic approach has been applied to KPE with encouraging results, and a few studies reported a lower rate of adhesions and bowel perforations after LT when the previous Kasai was performed through laparoscopy [27,28]. Nevertheless, this finding was not confirmed in other series [29] and the advantages of laparoscopy over open surgery in terms of clearance of jaundice and survival with native liver are still controversial [29,30].

This study has an inherent limitation, as there are no comparisons with other surgical techniques or with an historic cohort before 2010. Nevertheless, according to our 30 years of experience with pediatric liver transplantation, we found that in BA patients who have undergone previous standard KPE, the rate of intestinal adhesions and bowel perforations is much higher. A controlled trial is strongly advisable to test this hypothesis, and currently, one has been started in our Centre.

Our extended adhesion-sparing liver eversion during KPE can be considered an improvement of the standard techniques and could be included in the armamentarium of the KPE. As for the standard eversion technique, ASLE provides an excellent exposition of the portal plate, enabling both an accurate dissection of atretic extrahepatic bile ducts and portoenteroanastomosis. In addition, at transplantation, our technique, by reducing the formation of intra-abdominal adhesions, facilitates native liver total hepatectomy and decreases the intra-operative bleeding and the occurrence of bowel perforations.

## Figures and Tables

**Table 1:** Main outcomes of the series ^1^.

Number of patients	44
Median age at surgery, months (range)	13.1 (5–120)
Median weight at surgery, kg (range)	8.1 (5–23)
Median total transplant time	337 min (IQR = 272–371)
Median hepatectomy phase time	57 min (IQR = 50–67)
Median volume of PRBC transfusion	95 mL (IQR = 0–250)
Median volume of plasma transfusion	0 mL (IQR = 0–110)
Bowel perforation (surgical complication) - during LT; - after LT (1 month);	0 (0%) 0 (0%)
Mortality (1 year follow-up)	0 (0%)
Graft loss (1 year follow-up)	1 (2.2%)

^1^ Patients’ demographics and results of liver transplantation for failure of KPE. PRBC, packed red blood cells; LT, liver transplantation.

## Data Availability

The data presented in this study are available on request from the corresponding author.
